# Involvement of the Vitamin D Receptor in Energy Metabolism Revealed by Profiling of Lysine Succinylome of White Adipose Tissue

**DOI:** 10.1038/s41598-017-14477-8

**Published:** 2017-10-26

**Authors:** Han Su, Yan Lou, Yu Fu, Yalin Zhang, Ning Liu, Zuwang Liu, Yanyan Zhou, Juan Kong

**Affiliations:** 1 Department of Clinical Nutrition, Shengjing Hospital of China Medical University, Shenyang, 110004 China; 2 School of Fundamental Sciences, China Medical University, Shenyang, 110122 China; 3 Jingjie PTM Biolab (Hangzhou) Co.,Ltd., Hangzhou, 310018 China

## Abstract

Lysine succinylation, emerging as a novel post-translational modification, is closely related to the regulation of diverse biological processes, including many aspects of metabolism. Growing evidence suggests that low vitamin D status might exert an adverse impact on energy balance, adipogenesis and inflammation in white adipose tissue (WAT). However, whether there are any interactions between vitamin D and lysine succinylation still remains unknown. Here, combining high-affinity enrichment of lysine succinylated peptides with mass spectrometry and bioinformatics analysis, we reported the systematic profiling of the lysine succinylome, identifying 209 sites occurring on 159 proteins were up-regulated, 3 sites in 3 proteins were down-regulated in vitamin D receptor (VDR)^−/−^ mice. Bioinformatics analysis reveals potential impacts of lysine succinylation on diverse biological processes and molecular functions, especially on carbon biotransformation, fatty acid metabolism and TCA cycle. Furthermore, eight unique motifs surrounding the succinylation sites were validated. Collectively, our findings demonstrate the first comprehensive profiling of WAT succinylome in VDR^−/−^ mice, and provide crucial clues for further elucidating the underlying mechanisms of the involvement of the VDR in energy metabolism.

## Introduction

Post-translational modifications (PTMs) are one of the most fundamental and complex mechanisms involved in the regulation of diverse cellular events, such as gene expression, metabolism, protein synthesis, cell cycle, etc^1–3^. Lysine residue is a major site of PTMs because of its own spatial structural features. In addition to several common types of protein acetylation^4,5^, malonylation^6^, ubiquitination^7^, methylation^8^, lysine succinylation is a newly identified and validated modification as a novel PTM, where a succinyl group is transferred to a lysine residue^9–11^. Accumulating evidence suggests lysine succinylation is widely present in prokaryotes and eukaryotes, such as *E. coli* ^12,13^, yeast, *Mycobacterium tuberculosis* ^14^, *Toxoplasma gondii* ^15^, mouse hepatocytes and human cells^16^. In contrast to acetylation and methylation, lysine succinylation could induce a 100 Da change in mass. As a result, it can induce charge status to transform from +1 to −1 under physiological pH conditions, which in turn promotes dramatic structural and functional changes of a substrate protein^9^. Lysine succinylation is emerging as an essential regulator of mitochondrial proteins and energy metabolism, owing to its fundamental role in the process of tricarboxylic acid (TCA) cycle^17^. Furthermore, it is reported that the level of succinylation increases in tissues of mice in response to fasting conditions^16^. Additionally, increasing evidence suggests that lysine succinylation dramatically interacts with multiple enzymes in energy metabolism. Glyceraldehyde 3-phosphate dehydrogenase (GAPDH) and isocitrate dehydrogenase (IDH) are strongly associated with glycolytic pathway and TCA cycle, whereby it gives rise to elevated activity of glucose and fatty acid metabolism^9^. Pyruvate dehydronase (PDH), a critical metabolic enzyme involved in the generation of acetyl-CoA from pyruvate in TCA cycle, is also a target of lysine succinylation^16^. SIRT5, which catalyzes the desuccinylation of lysine residue in the consumption of NAD^+^, could facilitate urea cycle function by modulating carbamoyl phosphate synthase (CPS1) and purine metabolism *via* urate oxidase^18,19^. Consequently, lysine succinylation might play crucial roles in energy metabolism through modulating the activity of multiple metabolic enzymes.

Vitamin D receptor (VDR), a member of the nuclear receptor superfamily, is expressed almost ubiquitously. Upon ligand binding to 1, 25-dihydroxyvitamin D_3_, which is the active form of vitamin D, VDR forms a heterodimer with retinoic acid receptor (RXR) and translocates to nucleus, where it binds to vitamin D response element (VDRE) to mediate target gene transcriptions and expressions^20^. Mounting evidence suggests that besides regulating calcium homeostasis and maintaining skeletal health, vitamin D has a large spectrum of extraskeletal effects on multiple organs and metabolic processes, including cardiovascular^21^, respiratory^22^, renal and immune system^23,24^. Clinical and epidemiological studies demonstrate that low vitamin D status is closely linked with obesity^25^. Notably, vitamin D could directly modulate white adipose tissue (WAT) formation and function through mediating gene expressions. VDR^−/−^ mice tend to have lower WAT mass as a conspicuous whole-body phenotype. The size of adipocytes in VDR^−/−^ mice is smaller in comparison with wild-type (WT) mice^26^. In addition, the levels of serum leptin, insulin, triglyceride and blood glucose are lower in VDR^−/−^ mice than in WT mice. Conversely, food intake is higher than that of WT mice^26^. Wong and his coworkers have confirmed that the rate of fatty acid β-oxidation is elevated and gene expressions of uncoupling protein (UCP) 1, UCP2 and UCP3 are all drastically up-regulated in VDR^−/−^ mice, indicating that VDR knockout mice tend to have higher energy expenditure^27,28^. However, the mechanisms of the link between vitamin D deficiency and energy metabolism remain largely unknown. In addition, a great number of studies show that there is a strong interplay between vitamin D system and PTMs. The VDR/RXR dimer could interact with histone modifications, mainly acetylation, *via* regulating the expressions of histone acetyltransferases (HATs)^29^. The impact of vitamin D system on methylation has also been confirmed^30^. However, little is known whether there are any interactions between vitamin D and lysine succinylation.

Therefore, characterization of the lysine succinylome in the WAT in response to VDR might shed light on the impact of succinylation and VDR on energy metabolism. Here, based on an integrated proteome-wide method, we performed a systematic identification of the lysine succinylome in WAT from WT and VDR^−/−^ mice. In the present study, we identified a total of 543 lysine succinylation sites in 340 protein groups, among which 353 sites in 239 proteins were quantified with different cellular locations and biological functions. Compared to WAT in WT mice, 209 lysine succinylation sites in 159 proteins were up-regulated, 3 lysine succinylation sites in 3 proteins were down-regulated in VDR^−/−^ mice. Furthermore, eight unique motifs were validated by systematic bioinformatics analysis of the sequence flanking on each side of the succinylation sites. Taken together, our findings provide the first comprehensive profiling of WAT succinylome in WT and VDR^−/−^ mice.

## Results and Discussion

### Lysine hypersuccinylation proteome in WAT of VDR^−/−^ mice

**Figure 1:**
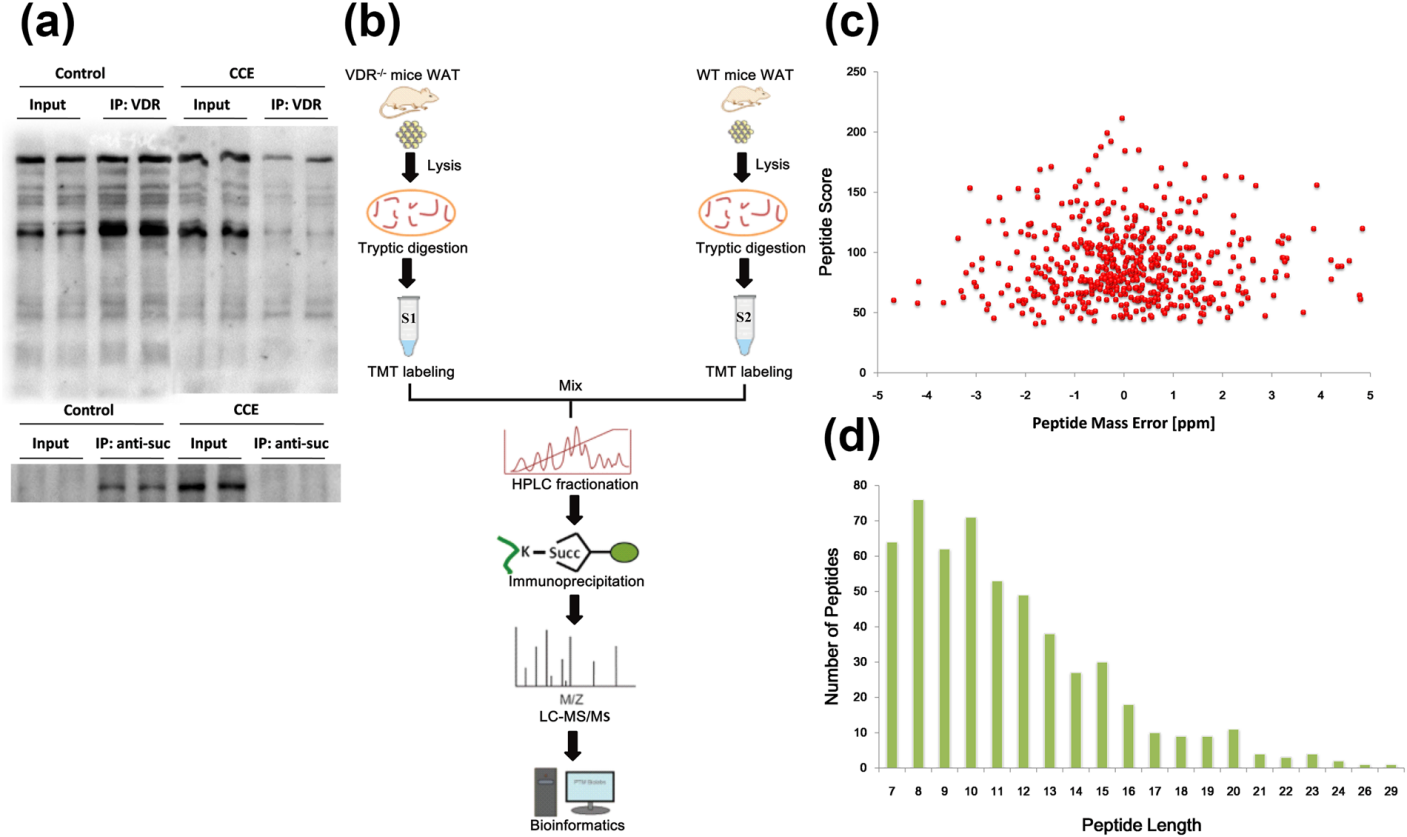
Profiling lysine succinylome in WT and VDR^−/−^ mice white adipose tissue (WAT). (**a**) Co-IP analysis of WT mice WAT using succinyl-lysine antibody demonstrates the presence of succinylated proteins. (**b**) Experiment Workflow for the identification of lysine Succinylation in WT and VDR^−/−^ mice WAT. (**c**) Peptide score of all succinylated peptides is plotted as a function of calibrated peptide mass errors measured from all identified peptides in parts-permillion (ppm). (**d**) Length distribution of the identified peptides.

**Figure 2:**
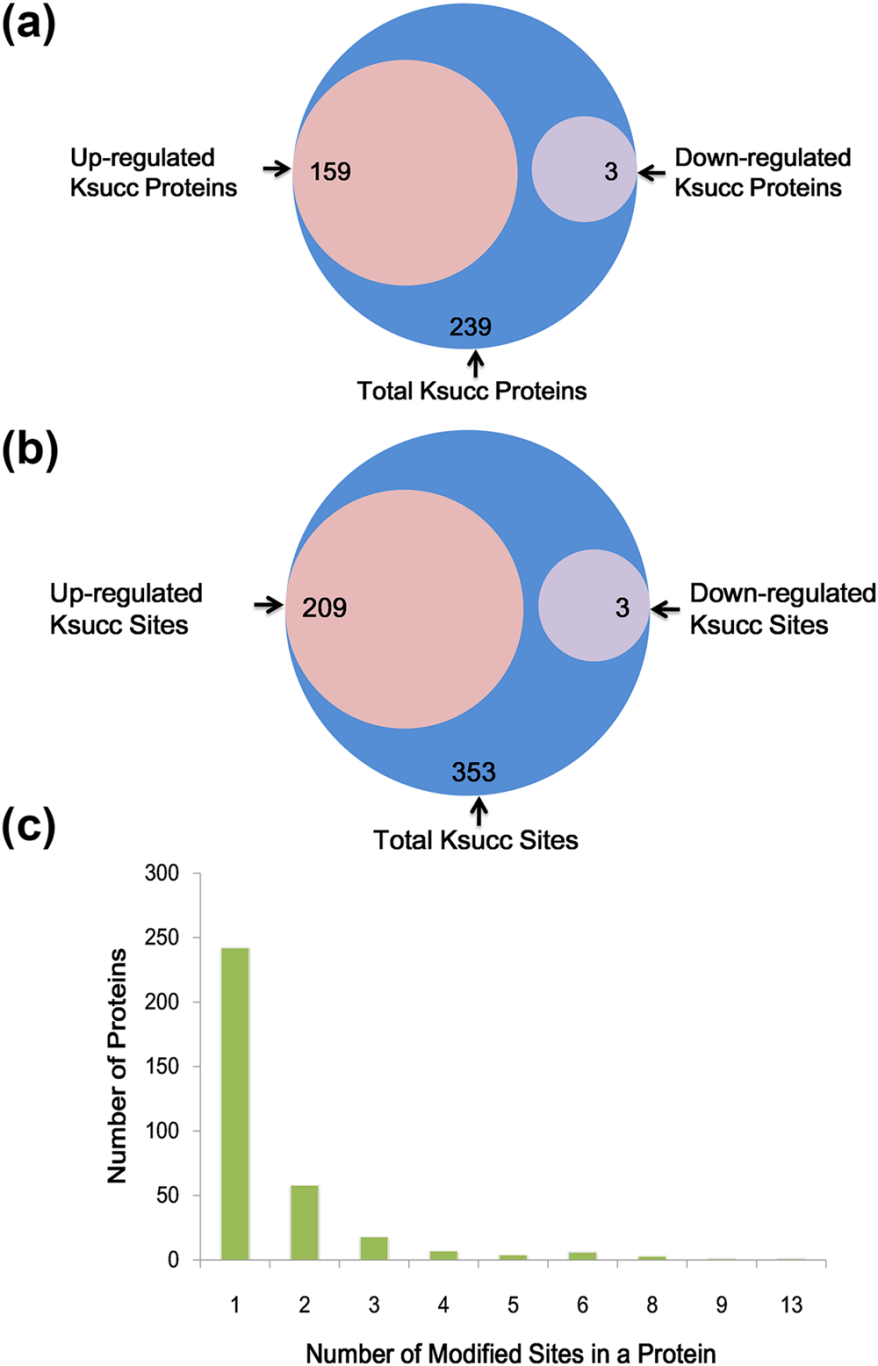
Quantification of succinylated proteins and sites. (**a**) Quantification of succinylated proteins. (**b**) Quantification of succinylated sites. (**c**) Distribution of succinylated proteins based on their number of succinylation peptides.

To determine if there is crosstalk between VDR and lysine succinylation, we carried out the co-immunoprecipitation (Co-IP) test to recognize succinyl-lysine pulled-down by VDR in WAT. The data demonstrated that there was less wide range of molecular masses succinylated in WAT of mice treated by cholecalciterol cholesterol emulsion (CCE), which was a precursor of 1,25(OH)_2_ vitamin D3 (Fig. 1a). In order to further determine the global succinylated protein and sites, the robust workflow by the integration of affinity enrichment, basic HPLC fractionation and LC-MS/MS analysis was adopted (Fig. 1b). The mass error was set to ±5 ppm for precursor ions (Fig. 1c). Additionally, the length of most identified peptides ranged from 7 to 23 (Fig. 1d). Based on the approach previously described, we identified a total of 543 lysine succinylation sites occurring on 340 proteins in WAT of WT and VDR^−/−^ mice with a peptide score over 40. To investigate whether the absence of VDR significantly impacted the global succinylation protein expression, we quantified succinylated protein levels in WT and VDR^−/−^ mice. Of all 340 succinylated proteins, 353 sites occurring on 239 proteins were quantified (Fig. 2a,b). In the present study, the quantitative ratio of lysine succinylation in WAT of WT and VDR^−/−^ mice over 2.0 was considered as up-regulation, whereas quantitative ratio below 1/2.0 (0.5) was regarded as down-regulation (P < 0.05). A number of differentially expressed proteins and lysine succinylation sites were obtained in each group. Eventually, we found 209 sites in 159 proteins were up-regulated, 3 sites in 3 proteins were down-regulated (Fig. 2a,b). Our data revealed that around 37.1 (59/159) succinylated proteins showed more than 4-fold change in abundance in WAT of VDR^−/−^ mice. Of the 340 identified succinylated proteins, 71.2% (242/340) proteins possessed a single succinylated site, 17.1 (58/340) proteins possessed two succinylated sites, 5.3% (18/340) possessed three succinylated sites (Fig. 2c). It is noteworthy that the most extensively succinylated protein was isocitrate dehydrogenase (IDH) in mitochondria, which was subjected to 13 independent succinylated lysine residues. Previous study reported that IDH, a critical metabolic enzyme involved in the TCA cycle, was also dramatically succinylated in mouse liver and Hela cells^16^.

Vitamin D signaling interacts with epigenetic regulatory system on multiple levels. VDR gene has large CpG islands in its promoter region, thereafter can be silenced by DNA methylation. Several studies have confirmed that 1, 25-D3 is able to alter DNA methylation in the promoter of some genes. Intriguingly, it was found that there was a weak correlation of vitamin D level with CpG islands (CGI) methylation and genomic long interspersed nuclear element-1 (LINE-1) methylation^30^. Another study suggested that high level of vitamin D intake was closely associated with lower methylation of WNT signaling antagonists^31^. Additionally, the liganded VDR could interact with histone acetyltransferases (HATs) to promote transcriptional activation^32^.

However, no clear picture has emerged on the role of lysine succinylation in vitamin D signaling. Interestingly, 35 histone lysine succinylation sites were found in histones^6^, whose succinylation level was mediated by SIRT 5^16^. Lysine succinylation sites mostly exist at the C-terminal globular domains^6^. Detection of lysine succinylation in histones, rather than in other nuclear proteins, indicates that similar with lysine acetylation, lysine succinylation might be regulated by vitamin D signaling through the alternations of histone modifications, which requires further investigation.

### Defining and characterizing the lysine succinylome in WAT

**Figure 3:**
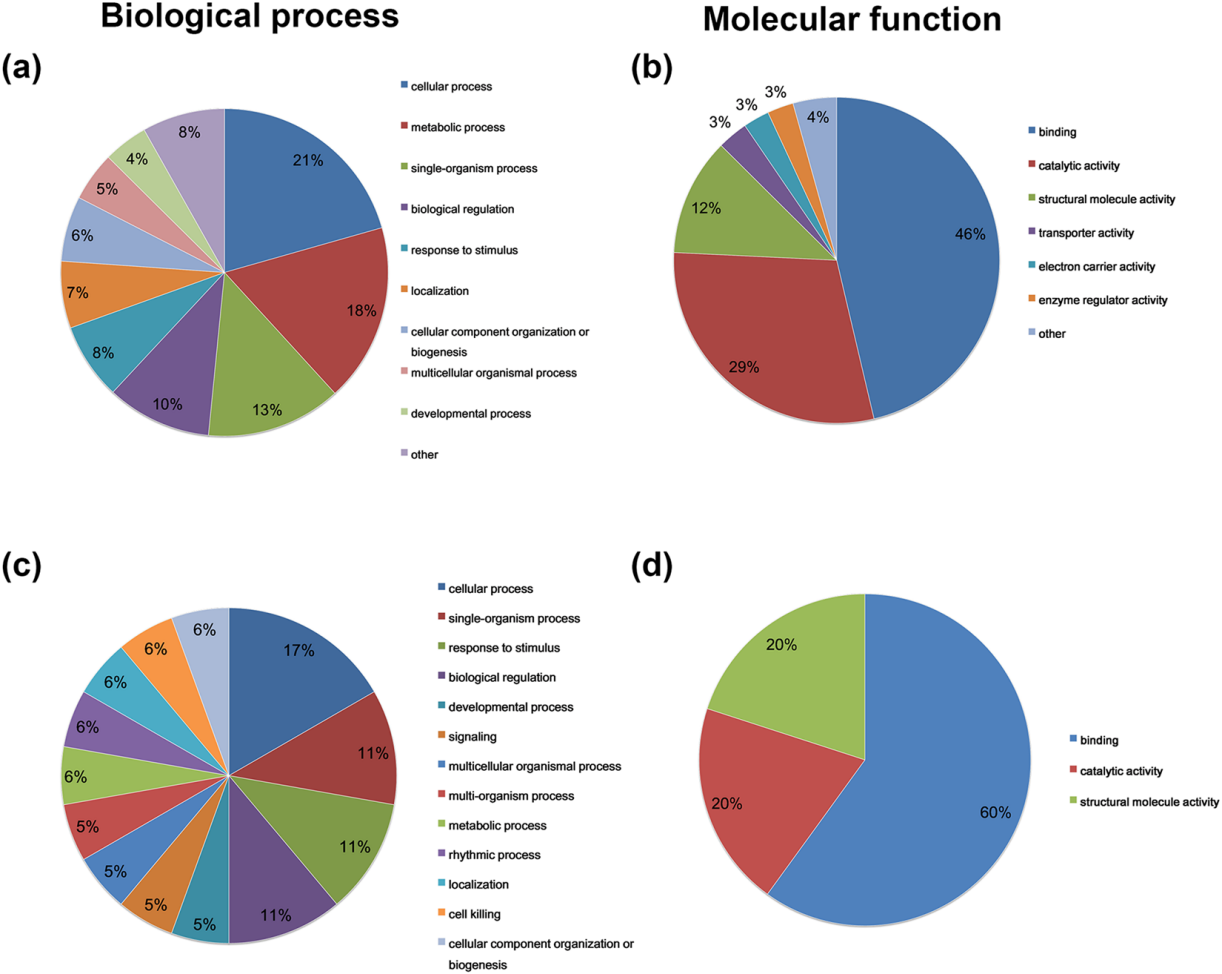
Gene ontology functional classification of the identified succinylation proteins. (**a**) The classification of the up-regulated succinylation proteins based on biological process. (**b**) The classification of the up-regulated succinylation proteins based on molecular functions. (**c**) The classification of the down-regulated succinylation proteins based on biological process. (**d**) The classification of the down-regulated succinylation proteins based on molecular functions.

To better understand the characterizations of succinylated proteins in WT and VDR^−/−^ mice WAT, we annotated all the validated succinylated proteins by the means of GO functional classification based on their biological process and molecular function (Fig. 3). The classification results in terms of biological process demonstrated that the largest class of succinylated proteins was enzyme proteins in association with cellular process, accounting for 21% and 17% of the all annotated succinylated proteins in the up-regulated group and down regulated group, respectively (Fig. 3a,c). Moreover, another large group involved in biological process was proteins associated with metabolic process, representing for 18% and 6% of the total succinylated proteins in the up-regulated group and down regulated group (Fig. 3a,c). Additionally, in terms of molecular function, the largest class of succinylated proteins was binding proteins, which accounted for 46% and 60% of all identified proteins in the up-regulated group and down-regulated group (Fig. 3b,d). The second largest class was comprised of proteins participated in catalytic activities, accounting for 29% and 20% of the all annotated succinylated proteins in the up-regulated group and down-regulated group. The GO analysis of the succinylome indicates that the succinylated proteins were strongly implicated in metabolic process and catalytic function, suggesting that lysine succinylation might play a certain role in energy metabolism modulated by vitamin D system.

**Figure 4:**
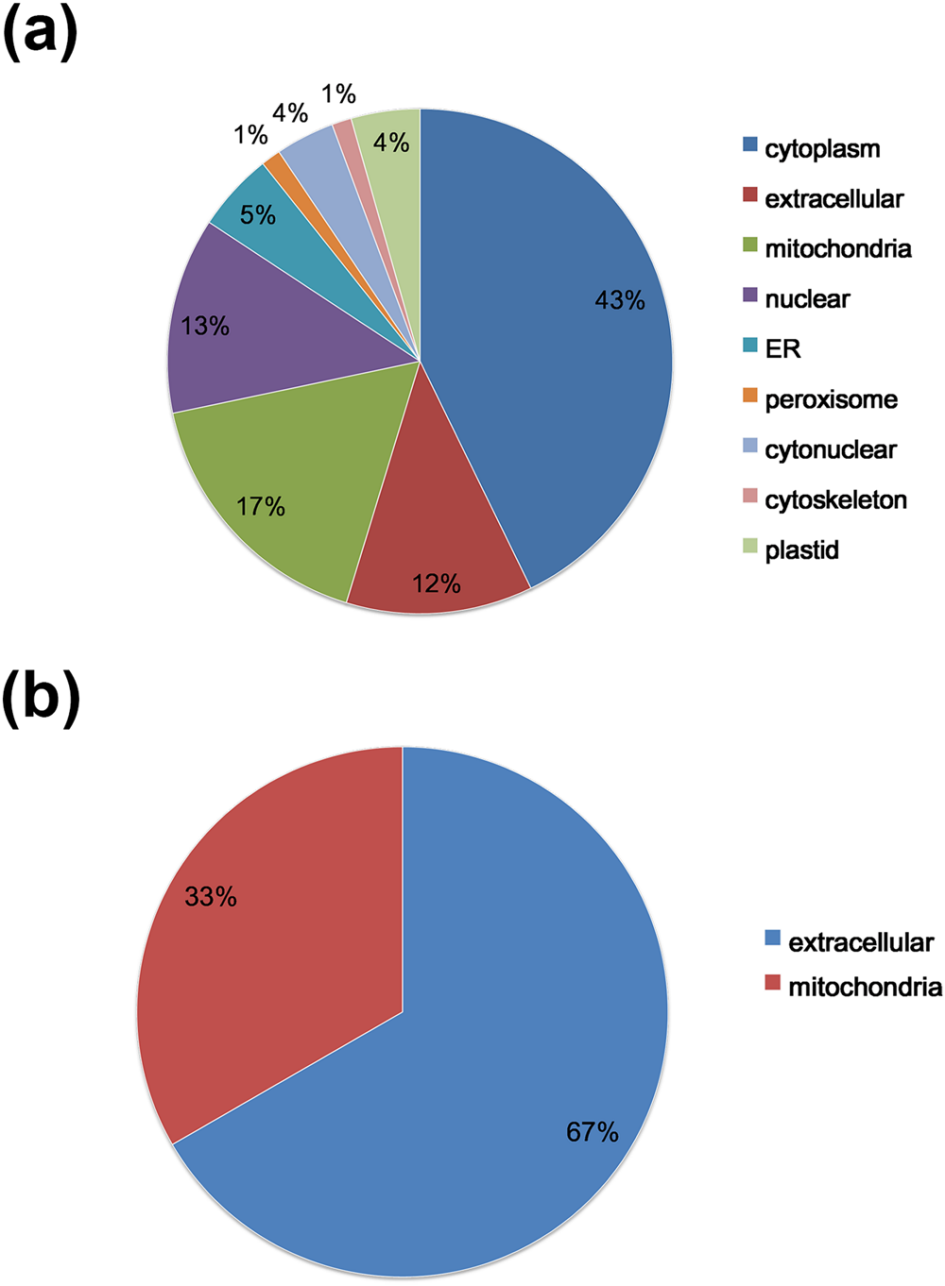
The GO annotation of the subcellular locations of the succinylation proteins. (**a**) The subcellular locations of the up-regulated succinylation proteins. (**b**) The subcellular locations of the down-regulated succinylation proteins.

Furthermore, we performed cellular compartment analysis to confirm the distribution of succinylated proteins. The classification results showed that the most up-regulated succinylated proteins were predicted to be in cytoplasm (43%). As expected, the second largest group of up-regulated succinylated proteins was located in mitochondria (17%). A few proteins are predicted to be in the nuclear (13%), extra cellular (12%), ER (5%), as shown in Fig. 4a. In contrast, of the 3 down-regulated succinylated proteins, two were distributed in extracellular, the other one was in mitochondria (Fig. 4b). Succinylation is largely enriched in the mitochondria, whereas some no-mitochondria proteins are also likely to be succinylated, such as ribosome and nuclear proteins^33^. Due to the fact that succinate is able to span the mitochondrial membrane and can be formed in the cytoplasm as a byproduct of α-ketoglutarate-dependent enzymes^34^, the occurrence of frequent lysine succinylation out of mitochondria in our study suggested that succinyl-CoA, succinate, or other succinyl-metabolites may facilitate succinylation in the cytoplasm and nucleus, which was consistent with previous studies^16^. More importantly, a huge proportion of mitochondrial proteins showed noticeably up-regulated lysine succinylation, suggesting potential roles of lysine succinylation in modulating energy balance in VDR^−/−^ mice.

### Enrichment analysis of the lysine succinylation in WAT

**Figure 5:**
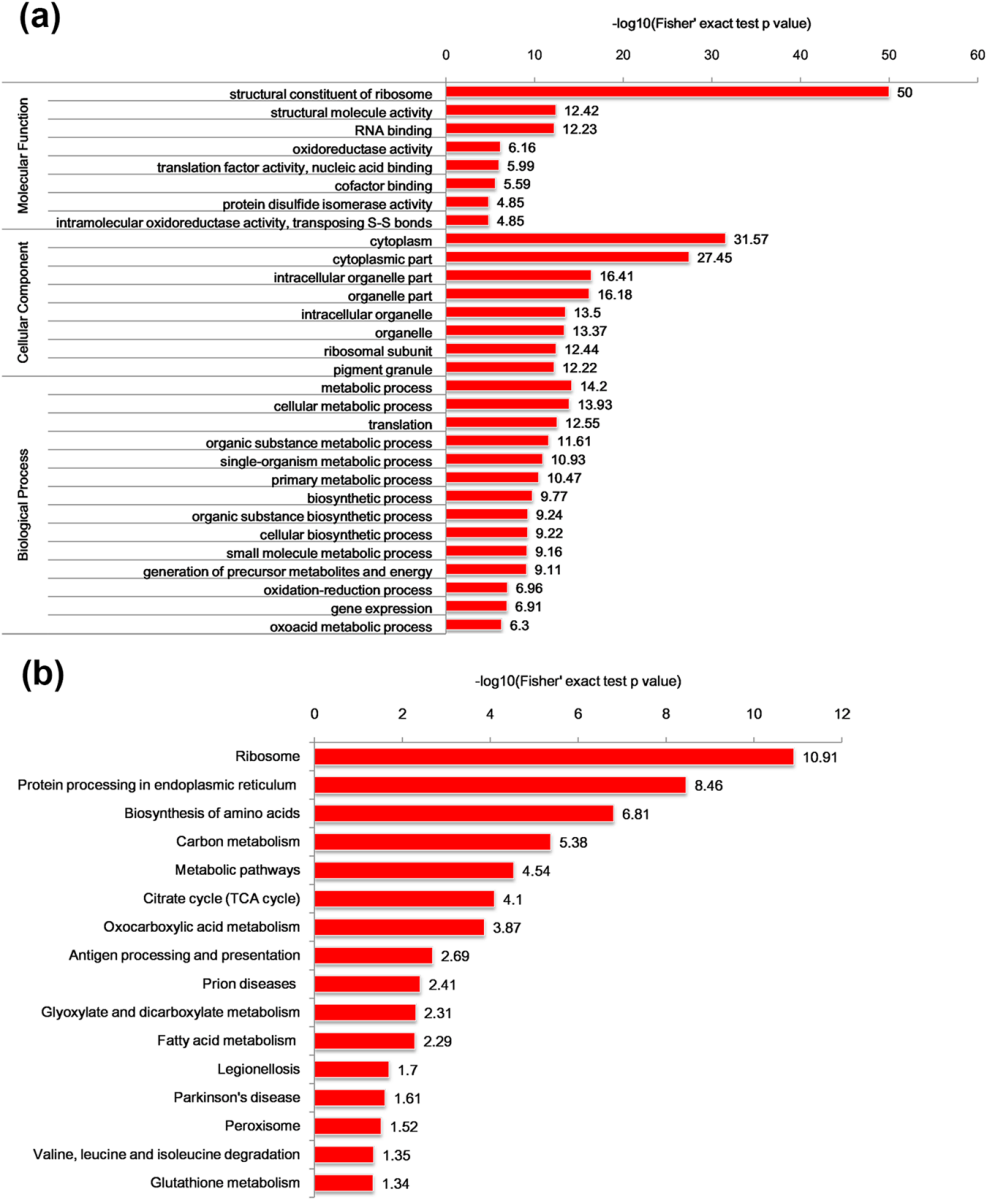
Enrichment analysis of the succinylation proteins. (**a**) GO enrichment analysis of the succinylation proteins. (**b**) KEGG pathway enrichment analysis of the succinylation proteins.

To determine the preferred functional categories of proteins in lysine succinylation, we carried out the GO enrichment analysis of the succinylation dataset in terms of three categories: molecular function, biological process and cellular component. Our data demonstrated that succinylated proteins in WAT of VDR^−/−^ mice were greatly enriched in metabolic and biosynthetic processes (Fig. 5a). From the GO enrichment analysis on molecular functions, we found that the up-regulated succinylated proteins were substantially enriched for binding activity and being structural constituent of ribosome (Fig. 5a). In the GO cellular component category, a tremendous proportion of the up-regulated succinylated proteins were distributed in the intracellular part, such as cytoplasm, intracellular organelle and organelle part, intracellular organelle and organelle (Fig. 5a). In the biological category, a wide range of metabolic process and biosynthetic process were observed to be the most enriched groups of the up-regulated succinylated proteins. On the contrary, the enrichment of the down-regulated succinylated proteins was located in the extracellular part and involved in binding functions.

To obtain a comprehensive view of the metabolic process involved, we further carried out KEGG enrichment analysis. The enrichment results illustrated that the most three enriched pathways were involved in ribosome, protein processing in endoplasmic reticulum and biosynthesis of amino acids. Additionally, multiple metabolic pathways were highly represented in carbon metabolism, metabolic pathways, TCA cycle, fatty acid metabolism, oxocarboxylic acid metabolism, glyoxylate and dicarboxylate metabolism, glutathione metabolism (Fig. 5b). Studies have revealed that lysine succinylation may get involved in the regulation of global cellular energy metabolism, because there are amounts of targets of succinylation in the proteins involved in cellular metabolism^16^. The activity of some enzymes involved in the glucose and lipid metabolism is up-regulated to meet cellular requirements for ATP production^16^. It is found that succinylation is essential for the regulation of enzymes in carbon metabolism in bacteria and human cells^16,35^. Our findings are in line with previous studies, indicating that proteins involved in metabolic pathways are subjected to succinylation.

### Motif analysis of identified lysine succinylated proteins in WAT

**Figure 6:**
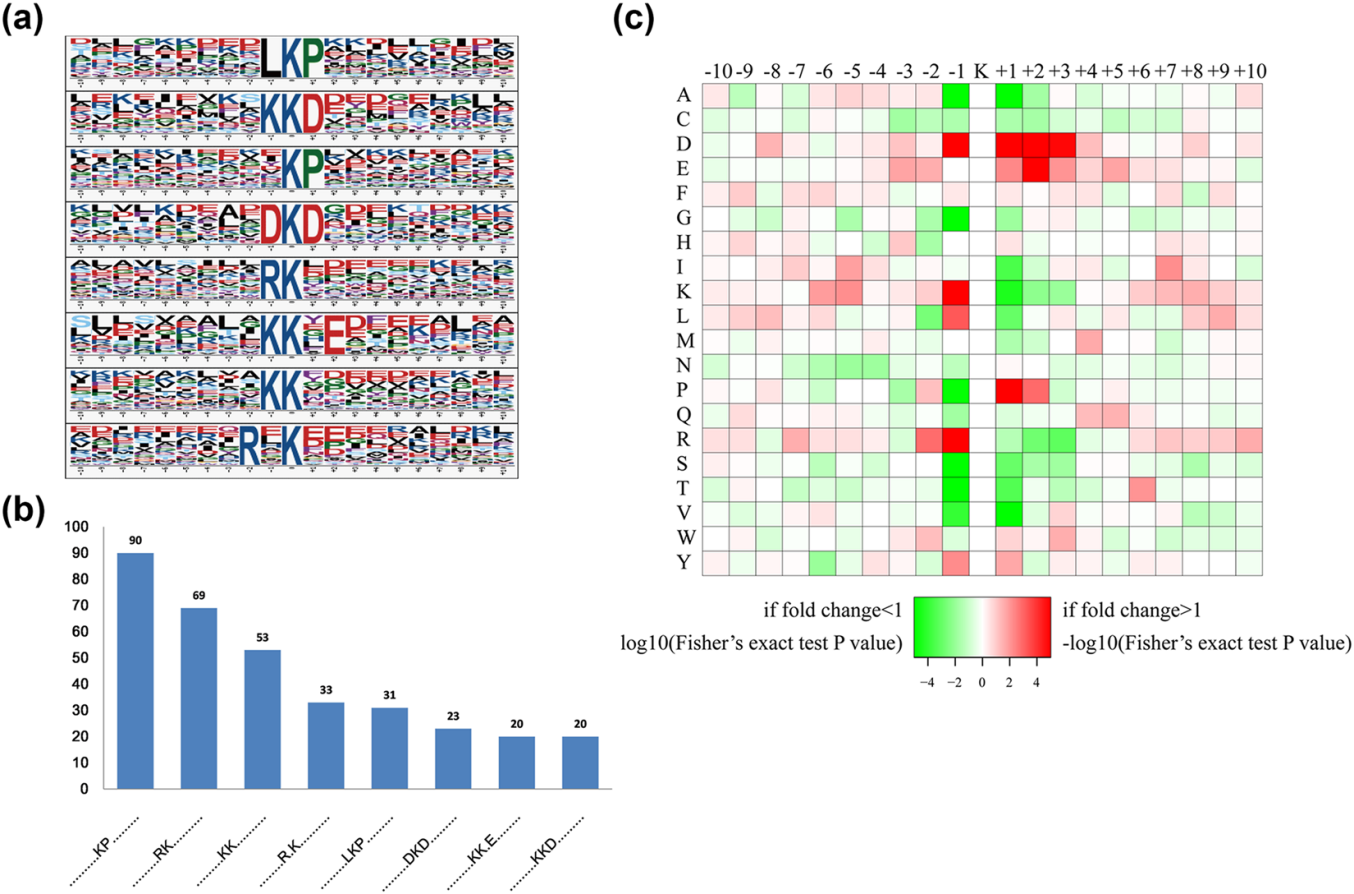
Properties of succinylated peptides. (**a**) Succinylation motifs and conservation of suucinylation sites. (**b**) Number of identified peptides in each conserved motif. (**c**) Heat map of the amino acid composition surrounding the lysine succinylation site showing the frequency of different types of amino acids. Red indicates enrichment and green indicates depletion.

To further assess the nature of the succinylated lysines in WAT of VDR^−/−^ mice, we used Motif-X software, which was used to extract overrepresented patterns from any sequence, to examine the amino acid sequences flanking the succinylation site. We have defined 8 conserved succinylation site motifs on 339 unique sites, accounting for 62.4% (339/543) of identified sites in accordance with the specific amino acid sequence located 10 amino acids upstream and downstream of the succinylated sites (Fig. 6a,b). Furthermore, to determine if there is noticeable frequency of specific amino acids flanking the succinylation site, these results were further analyzed and demonstrated by heat map. The data showed a preference of Asp or Pro at the +1 position, Leu, lys, Arg or Asp at the −1 position, Glu at the +2 position and Arg at the −2 position (Fig. 6c). Consequently, we have identified LKP, KKD, KP, DKD, RK, KKXE, KK, RXK motifs as significantly overrepresented for lysine succinylation through motif analysis (X indicates a random amino acid residue), suggesting their crucial role in sequence motifs surrounding succinylation sites.

### A number of succinylated proteins engaged in metabolic pathways

**Figure 7:**
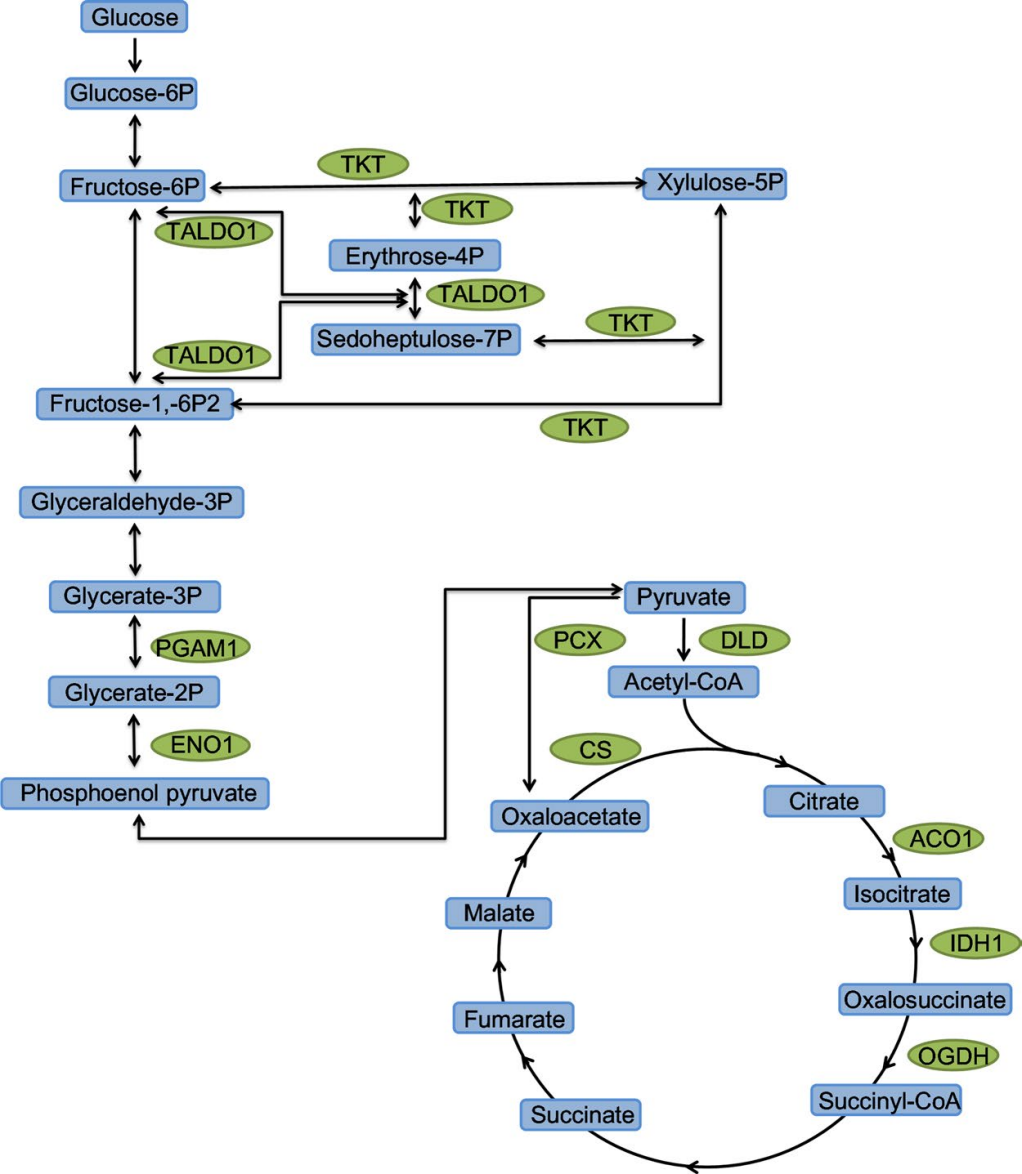
Succinylated enzymes involed in carbon metabolic and TCA cycle pathways. Green column indicates up-regulated succinylated enzyme. Transketolase: TKT, transaldolase 1:TALDO1, phosphoglycerate mutase 1: PGAM1, enolase 1: ENO 1, dihydrolipoamide dehydrogenase: DLD, pyruvate carboxylase: PCX, citrate synthase: CS, aconitate hydratase: ACO1, isocitrate dehydrogenase: IDH, oxoglutarate dehydrogenase: OGDH.

Pathway analysis results showed that 68 up-regulated succinylated proteins participated in a variety of metabolic pathways, which include carbon metabolism, TCA cycle, fatty acid metabolism, etc. conversely, there was no down-regulatedly succinylated proteins involved in any pathway. Firstly, we found several key enzymes were succinylated in the carbohydrate metabolism and TCA cycle pathway (Fig. 7), including transketolase (TKT), transaldolase 1 (TALDO1), phosphoglycerate mutase 1 (PGAM1), enolase 1 (ENO 1), dihydrolipoamide dehydrogenase (DLD), pyruvate carboxylase (PCX), citrate synthase (CS), aconitate hydratase (ACO1), isocitrate dehydrogenase (IDH), oxoglutarate dehydrogenase (OGDH) (Fig. 7). For instance, IDH1, which is a key rate-limiting step enzyme, catalyzes the third step of the TCA cycle in conversing isocitrate to α-ketoglutarate and carbon dioxide. We have identified 13 succinylation sites on IDH, which has the most extensive succinylated sites of all succinylated proteins. Previous studies have identified several succinylation sites on IDH in *E. coli* ^9^. CS is also a critical enzyme modulating the activity of TCA cycle.

**Figure 8:**
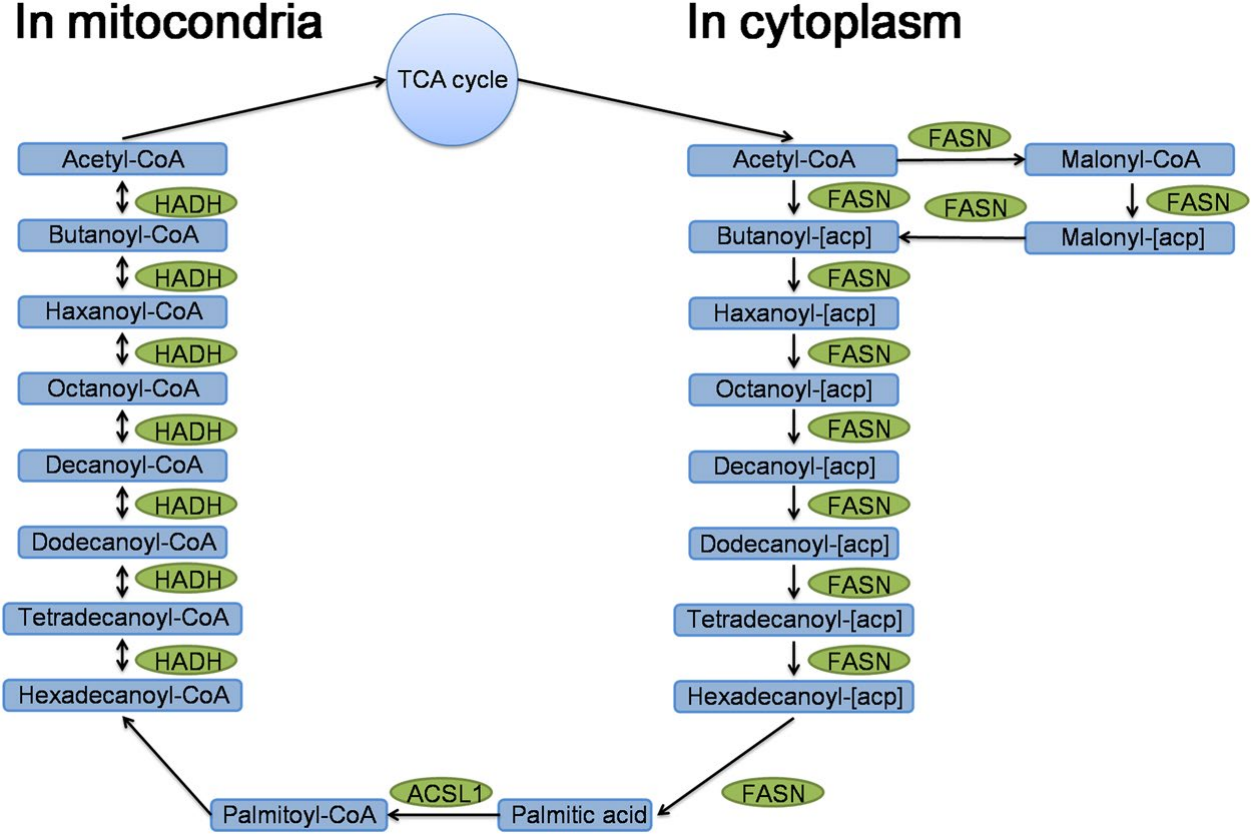
Succinylated enzymes involved in fatty acid metabolism pathways. Green column indicates up-regulated succinylated enzyme. fatty acid synthase: FASN, acyl-CoA synthetase long-chain family member 1: ACSL1, hydroxyacyl-Coenzyme A dehydrogenase: HADH.

Moreover, we found three crucial enzymes were up-regulatedly succinylated in fatty acid metabolism pathway in mitochondria and cytoplasm, which were fatty acid synthase (FASN), acyl-CoA synthetase long-chain family member 1 (ACSL1), hydroxyacyl-Coenzyme A dehydrogenase (HADH) (Fig. 8). Previous studies have also confirmed that FASN are succinylated in pathogenic *Mycobacterium tuberculosis*, thereby impacting fatty acid biosynthesis pathway^14^. Therefore, our findings suggested that lysine succinylation may play a central role in the TCA cycle and the fatty acid metabolism pathway.

In conclusion, through high-affinity enrichment of lysine succinylated peptides with mass spectrometry and bioinformatics analysis, for the first time, we provide a global profiling of the lysine succinylome in WAT of WT and VDR^−/−^ mice. We identified a total of 543 lysine succinylation sites occurring on 340 proteins, quantified 353 sites in 239 proteins. In comparison of the succinylation in WT mice, we found 209 sites in 159 proteins were up-regulated, 3 sites in 3 proteins were down-regulated in VDR^−/−^ mice. Moreover, extensive characterization of succinylated proteins further indicates that succinylation may play essential roles in a broad spectrum of physiology process ranging from biological processes, molecular functions, catalytic activity and as well as being distributed in different cellular compartment. In particular, several key enzymes were succinylated in metabolic pathways, including carbon metabolism, TCA cycle and fatty acid metabolism, which might account for the explanation of the impacts of VDR on energy metabolism. Taken together, our data provide novel insights into the role of lysine succinylation in WAT of WT and VDR^−/−^ mice, thereafter, elucidating lysine succinylation in the context of energy metabolism in the loss of VDR, which provide valuable clues for exploring the underlying mechanisms of the impact of VDR on energy metabolism in further studies.

## Methods

### Animals

The WT and VDR^−/−^ mice on a C57BL/6 background were generated through breeding of heterozygous mice. The genotyping of the mice was performed by PCR using mice tail samples. All experiment protocols in our study were approved by Animal Experiment Committee Shengjing Hospital of China Medical University, Shenyang, China. The approval number was 2017PS267K. We confirm that all experiments procedures were carried out in accordance with the guidelines established by the Animal Experiment Committee Shengjing Hospital of China Medical University, and every effort was made to minimize suffering. Male mice aged 8–10 weeks were enrolled in this study and sacrificed, and the WAT was harvested and snap frozen for succinylome analysis.

Male WT mice on the ICR background (aged 7–8 weeks old), were randomly divided into a control group (n = 5) and cholecalciterol cholesterol emulsion (CCE) group (n = 5). The CCE group was treated by supplying CCE in the drinking water (CCE: water = 10 μl: 100 ml) for 2 weeks. After the sacrifice of the mice, the WAT was harvested and stored at −80 °C for Co-immunoprecipitation (Co-IP) analysis.

### Protein extraction and digestion

The samples of WAT were sonicated with 12 short bursts of 10 s, followed by intervals of 10 s on ice for three times using a high intensity ultrasonic processor (Scientz) in lysis buffer (8 M urea, 1% Triton-100, 10 mM DTT and 0.1% Protease Inhibitor Cocktail III). Unbroken debris was removed by centrifugation for 10 min at 4 °C at 20 000 g. The protein was precipitated with cold 15% TCA for 2 h at −20 °C. The supernatant was discarded after centrifugation at 4 °C for 10 min. The resulting precipitate was washed with cold acetone for three times. The protein was re-suspended in buffer (8 M urea, 100 mM TEAB, pH 8.0). Afterwards, the protein concentration was measured with 2-D Quant kit (GE Healthcare) in accordance with the manufacturer’s instructions. The digested peptides were reduced with 10 mM DTT for 1 h at 37 °C. Reduced peptides were then alkylated with 20 mM IAA for 45 min at room temperature in darkness. For trypsin digestion, the protein sample was diluted by adding 100 mM TEAB to urea concentration less than 2 M. Eventually, trypsin was added at 1:50 trypsin-to-protein mass ratio for the first digestion overnight and 1:100 trypsin-to-protein mass ratio for a second 4-hour-digestion.

### Co-IP analysis

VDR and succinylated proteins were precipitated from WAT by adding anti-VDR or ies in WT mice. The antibody of anti-VDR (sc-1008) was purchased from Santa Cruz Biotechnology (CA, USA). The anti-succinyl-lysine antibody was obtained from PTM Biolabs. After incubation in blocking buffer at 1:1000 for 4 h at 4 °C, 10 μl of protein A-Sepharose beads were added, and incubated with moderate stirring at 4 °C for 12 h. The beads were then washed, and the immunoprecipitated protein complex was loaded into 10% SDS-PAGE gel, electrophoresed and processed for western blotting. To identify a molecular interaction between VDR and succinyl-lysine, the VDR-immunoprecipitating complex was blotted for succinyl-lysine, and succinyl-lysine -immunoprecipitating complex blotted for VDR.

### TMT labeling

After trypsin digestion, peptides were desalted by Strata X C18 SPE column (Phenomenex) and vacuum-dried. Peptides were reconstituted in 0.5 M TEAB and processed according to the manufacturer’s protocol for 6-plex TMT kit. Briefly, one unit of TMT reagent (defined as the amount of reagent required to label 100 μg of protein) were thawed and reconstituted in 24 μl ACN. The peptide mixtures were then incubated for 2 h at room temperature and pooled, desalted and dried by vacuum centrifugation. Samples isolated from WT and VDR^−/−^ mice were labeled respectively.

### Enrichment of succunylated lysine peptides

Immunoprecipitation was employed to enrich Ksucc peptides. Briefly, tryptic peptides were dissolved in NETN buffer (100 mM NaCl, 1 mM EDTA, 50 mM Tris-HCl, 0.5% NP-40, pH 8.0) and incubated with pre-washed antibody beads (PTM Biolabs) at 4 °C overnight with gentle shaking. After incubation, the supernatant was discarded and the beads were washed four times with NETN buffer and twice with ddH_2_O. The bound peptides were eluted from the beads with 0.1% TFA. The eluted fractions were combined and vacuum-dried. The remaining peptides were rinsed with C18 ZipTips (Millipore) according to the manufacturer’s protocols, followed by LC-MS/MS analysis.

### LC-MS/MS analysis

The immunoprecipitated peptides were redissolved in 0.1% FA, directly loaded onto a reversed-phase pre-column (Acclaim PepMap 100, Thermo Scientific). Peptide separation was carried out by a reversed-phase analytical column (Acclaim PepMap RSLC, Thermo Scientific) with an gradient increase from 7% to 18% solvent B (0.1% FA in 98% ACN) for 16 min, 18% to 22% for 8 min, 22% to 35% for 8 min and climbing to 80% in 5 min then holding at 80% for the last 3 min, all at a constant flow rate of 280 nl/min on an EASY-nLC 1000 UPLC system. The resulting peptides were analyzed by Q ExactiveTM Plus hybrid quadrupole-Orbitrap mass spectrometer (ThermoFisher Scientific).

The resulting peptides were subjected to a nanospray-ionization (NSI) source followed by tandem mass spectrometry (MS/MS) in Q ExactiveTM Plus (Thermo Scientific) coupled online to the UPLC. Intact peptides were detected in the Orbitrap at a resolution of 70 000. Peptides were selected for MS/MS using NCE setting as 30; ion fragments were detected in the Orbitrap at a resolution of 17 500. A data-dependent procedure that alternated between one MS scan followed by 20 MS/MS scans was applied for the top 20 precursor ions above a threshold ion count of 1E4 in the MS survey scan with 15.0 s dynamic exclusion. The electrospray voltage applied was 2.0 kV. Automatic gain control (AGC) was used to prevent overfilling of the ion trap; 5E4 ions were accumulated to generate MS/MS spectra. For MS scans, the m/z scan range was 350 to 1800. Fixed first mass was set as 100 m/z.

### Database search

The protein and succinylation sites were identified using MaxQuant with integrated Andromeda search engine (v.1.4.1.2). Tandem mass spectra were searched against Swissprot_mousedatabase (16,717 sequences) concatenated with reverse decoy database. Trypsin/P was specified as cleavage enzyme allowing up to 4 missing cleavages, 4 modifications per peptide and 5 charges. Mass error was set to 10 ppm for precursor ions and 0.02Da for fragment ions. Carbamidomethylation on Cys was specified as fixed modification and oxidation on Met, succinylation on Lys and acetylation on protein N-terminal were specified as variable modifications. False discovery rate (FDR) thresholds for protein, peptide and modification site were specified at 1%. Minimum peptide length was set at 7. All the other parameters in MaxQuant were set to default values. The site localization probability was set as >0.75.

### Bioinformatics analysis

#### Protein annotation

Gene Ontology (GO) annotation proteome was carried out through the UniProt-GOA database (http://www.ebi.ac.uk/GOA/). Firstly, Converting identified protein ID to UniProt ID and then mapping to GO IDs by protein ID. If some identified proteins were not annotated by UniProt-GOA database, the InterProScan soft would be used to annotated protein’s GO functional based on protein sequence alignment method. Then proteins were classified by GO annotation based into three categories: biological process, cellular component and molecular function.

#### KEGG pathway annotation

Kyoto Encyclopedia of Genes and Genomes (KEGG) database was used to annotate protein pathways. KAAS, a KEGG online service tool, was used to annotate protein’s KEGG database description. Then, the annotation result was mapped to the KEGG pathway database using KEGG online service tools KEGG mapper.

#### GO/KEGG pathway functional enrichment analysis

For enrichment or depletion (right-tailed test) of specific annotation terms among the members of resulting protein clusters, Fisher’s exact test was used to obtain the p values. In all of the clusters, any terms with p values below 0.05 were treated as significant.

#### Analysis of sequence model and succinylation sites

The sequence models consisted of amino acids at specific positions of the succinyl-21-mers (10 amino acids upstream and downstream of the succinylation sites) in all protein sequences surveyed using Motif-x. Meanwhile, the database of mice protein sequences was used as background database parameter, and other parameters were set as default.

#### Motif-based clustering analysis

All lysine succinylation substrate categories obtained after enrichment were collated along with their p values and then filtered for categories that were at least enriched in one of the clusters with p value < 0.05. This filtered p-value matrix was transformed using the function x = −log 10 (p value). Finally, these x values were z transformed for each category. Subsequently, these z scores were clustered by one-way hierarchical clustering (Euclidean distance, average linkage clustering) in Genesis.

## Electronic supplementary material

Supplementary Information

Dataset 1

Dataset 2

Dataset 3
