# Supplementary material for: Involvement of the Vitamin D Receptor in Energy Metabolism Revealed by Profiling of Lysine Succinylome of White Adipose Tissue
Source: Sci Rep. 2017 Oct 26;7:14132. doi: 10.1038/s41598-017-14477-8 (PMC5658373; doi:10.1038/s41598-017-14477-8)
Supplement: Supplementary file 1 — Supplementary Information [file 41598_2017_14477_MOESM1_ESM.pdf]

Involvement of the Vitamin D Receptor in Energy Metabolism Revealed by Profiling of Lysine Succinylome of White Adipose Tissue

Han Su[1], Yan Lou[2], Yu Fu[1], Yalin Zhang[1], Ning Liu[1], Zuwang Liu[1], Yanyan Zhou[3], Juan Kong[1]*

[1]Department of Clinical Nutrition, Shengjing Hospital of China Medical University, Shenyang 110004, China

[2]School of Fundamental Sciences, China Medical University, Shenyang 110122, China

[3]Jingjie PTM Biolab (Hangzhou) Co.,Ltd., Hangzhou 310018, China

Corresponding Author

Juan Kong. E-mail: kongj1@sj-hospital.org.

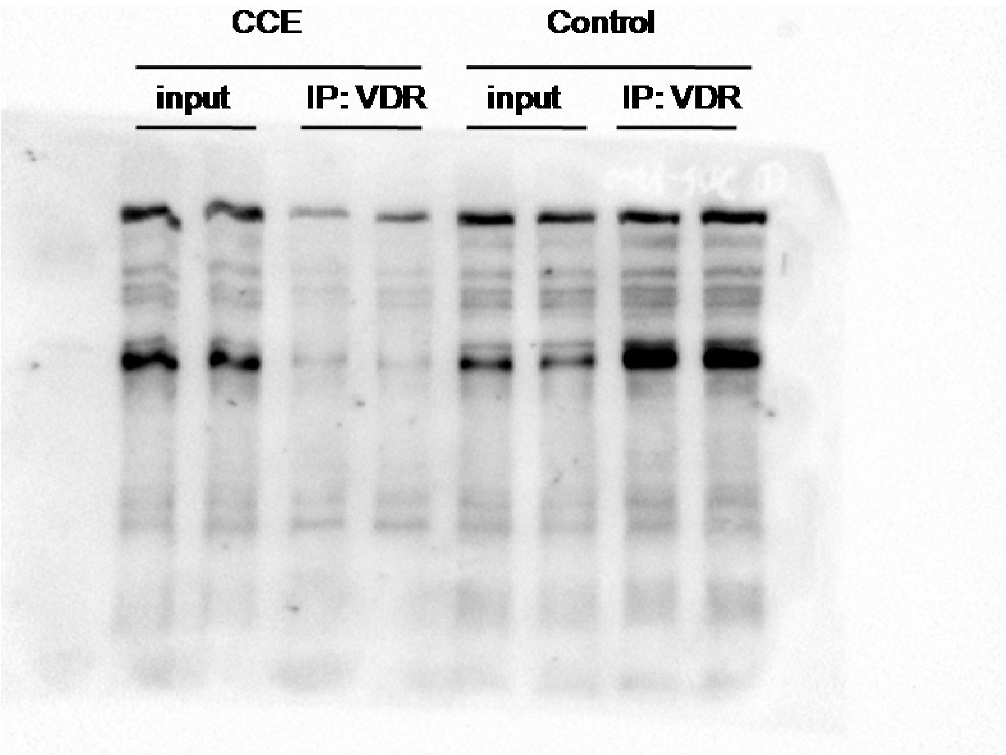
CCE
Control
input
IP: VDR
input
IP: VDR

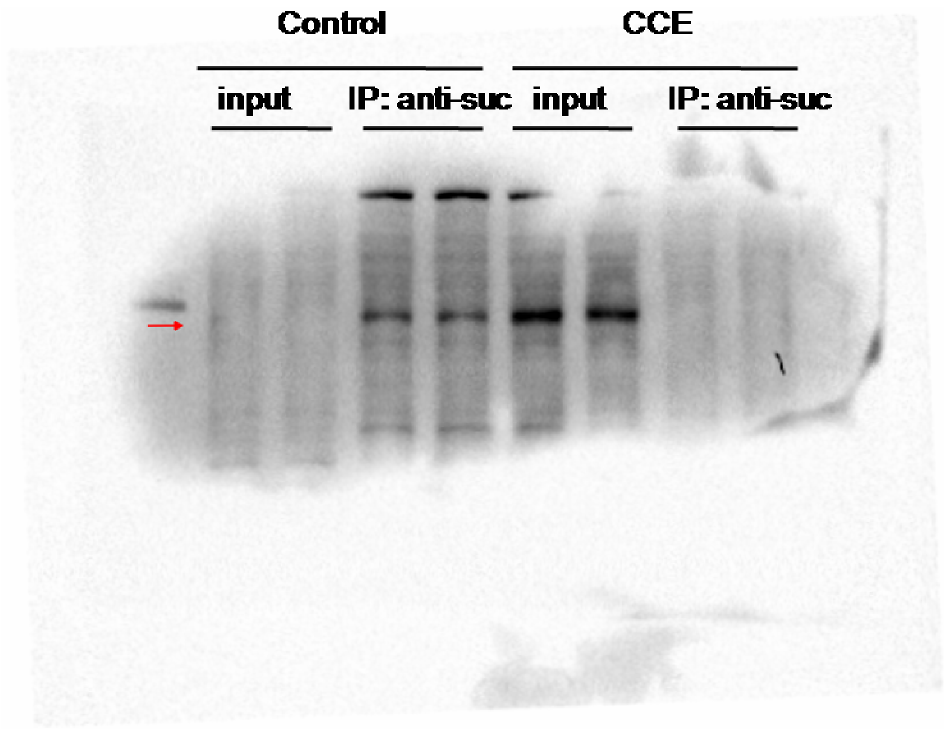
Control
CCE
input
IP: anti-suc
input
IP: anti-suc
